# Improved delivery of natural alkaloids into lung cancer through woody oil-based emulsive nanosystems

**DOI:** 10.1080/10717544.2018.1474970

**Published:** 2018-06-11

**Authors:** Jing Zhao, Shan Liu, Xueyuan Hu, Yunmei Zhang, Shenglei Yan, Hua Zhao, Mei Zeng, Yao Li, Lan Yang, Jingqing Zhang

**Affiliations:** a Chongqing Research Center for Pharmaceutical Engineering, Chongqing Medical University, Chongqing, China;; b Nursing College, Chongqing Medical University, Chongqing, China

**Keywords:** Woody oil-based nanosystem, efficient delivery, sensitivity increment, natural alkaloid, evodiamine

## Abstract

Most antitumor ingredients found in nature have poor solubility. These ingredients are expected to have much better absorption and higher bioavailability than synthetic antitumor agents. Woody oil emulsive nanosystems carrying poorly soluble natural alkaloids were fabricated (evodiamine (EA) carried by fructus bruceae oil-based emulsive nanosystems, or EFEN). Fructus bruceae oil has two excipient-like properties (oil phase and stabilizer) that contribute to the formulation and one drug-like property (antitumor effects) that synergizes with the antitumor effect of EA. The properties of EFEN were compared with free EA, a blank nanoemulsion, an EA-loaded emulsive nanosystem, and a fructus bruceae oil-loaded emulsive nanosystem. For the first time, this suggests that increases in the sensitivity of lung cancer cells to poorly soluble natural alkaloids can be achieved by delivering drugs using woody oil-based emulsive nanosystems. In this study, woody oil-based emulsive nanosystems efficiently deliver poorly soluble natural alkaloids.

## 1. Introduction

Natural alkaloids, including a quinolone alkaloid (evodiamine (EA), isolated from the unripened fruit of *Evodia rutaecarpa*) (Tan & Zhang, 2016), a chromone alkaloid (rohitukine, extracted from the stem of *Dysoxylum binectariferum*) (Safia et al., 2015) and piperidine alkaloids ((−)-cassine and (−)-spectaline, derived from a flower of *Senna spectabilis*) (Pereira et al., 2016), have been reported to have cytotoxic properties against different cancer cells. The antitumor activities of these alkaloids could be improved by synthesizing their derivatives (e.g. synthesis of piperidine alkene-alkaloids) (Kankala et al., 2014) or by being loaded with nanocarriers (e.g. dendrosomal solanine) (Mohsenikia et al., 2014).

Each alkaloid has a certain anticancer spectrum. To date, most efforts have focused on improving the known anticancer activities of alkaloids, and little research has attempted to broaden the anticancer spectrum of any alkaloid. Our team presented the first report that EA exhibits obvious cytotoxic effects on small-cell lung cancer (SCLC) cells (Fang et al., 2014). Moreover, EA showed low toxicity towards normal human peripheral blood cells but was cytotoxic to cancer cells, and it had no effect on body weight, in contrast to the serious weight loss caused by the clinically drug cisplatin (Tan & Zhang, 2016).

Among all cancers, lung cancer not only has the highest death rate but also has a low 5-year survival rate (＜15%). Standard chemotherapy treatment produces a clinical response but is far from satisfactory. Extensive research has focused on either improving the efficiency of current anti-cancer agents or discovering new chemical entities in nature. Considering that over 85% of lung cancers belong to the non-small-cell lung cancer (NSCLC) family, the effects of EA on NSCLC cells were further investigated. However, our preliminary study indicated that A549 cells were not sensitive to 20 μM EA over 72 h (EA was prepared as a suspension due to its very low solubility) (Tan et al., 2012). In light of recent reports that nanotechnology can increase the antitumor efficiency of drugs on chemo-resistant cells (which might be regarded as insensitive cells to some extent) (Lin et al., 2012), we speculated that nanotechnology may improve the sensitivity of lung NSCLC A549 cells to EA carried by suitable nanocarriers.

In recent years, emulsive nanosystems have been utilized to enhance the bioactivities of pharmaceuticals, supplements, and nutraceuticals (Aboalnaja et al., 2016). In particular, specific vegetable oil-based emulsive nanosystems have played wide roles in antimicrobial therapy (Franklyne et al., 2016; Li et al., 2016) and occasionally been used to enhance cytotoxicity in tumor cells (Desai et al., 2008). These specific vegetable oils, such as pine-nut oil (a woody plant seed oil) and tea tree oil (a woody plant leaf oil, also an essential oil), were used not only as an oil phase but also for their antioxidant activity, which increases the stability of the formulation since they have high concentrations of essential polyunsaturated fatty acids. In addition to serving as an oil phase and stabilizing agent, woody oils such as fructus bruceae oil (a woody plant seed oil) have a third function: supplying synergistic anti-tumor effects. Based on effectiveness and safety assessments of the addition of traditional Chinese medicines to chemotherapy or radiotherapy treatments for cancers, some specific vegetable oils are promising for clinical applications (Chen et al., 2016).

In this study, an woody oil emulsive nanosystem carrying a poorly soluble natural alkaloid (EA carried by fructus bruceae oil-based emulsive nanosystems, or EFEN) was fabricated and evaluated. The properties of EFEN were compared with free EA, a blank nanoemulsion (BNE), an EA-loaded emulsive nanosystem (EEN), and a fructus bruceae oil-loaded emulsive nanosystem (FEN). Our study showed that EFEN exhibited anti-tumor activities in NSCLC A549 cells while free EA did not. Moreover, the anti-tumor activity of EA was mediated by the inhibition of cell viability, the induction of apoptosis, and cell cycle arrest, among other effects. The increase in sensitivity may be related to enhanced uptake of EFEN by tumor cells. The pharmacokinetic behavior of EFEN was also different. For the first time, this suggested that increases in the sensitivity of lung cancer cells to poorly soluble natural alkaloids can be achieved by using woody oil-based emulsive nanosystems to delivering drugs. EFEN has the potential to be an effective, systemic anti-tumor treatment. This study shows that essential oil-based emulsive nanosystems can deliver natural alkaloids efficiently.

## 2. Materials and methods

### 2.1. Materials

All animal experiments were performed in accordance with the protocol approved by the Laboratory Animal Committee, Chongqing Medical University. EA was obtained from Yuancheng Technology Development Corporation (Wuhan, China). Phospholipid (Lipoid S 100) was provided from Lipoid GmbH (Ludwigshafen, Germany). Fructus bruceae oil was purchased from Kexin Biology Engineering Corporation (Shanghai, China). Ethyl oleate was purchased from Shanghai Chemical Corporation (Shanghai, China). Castor oil polyoxyethylene (35) ether (CPE) was obtained from BASF Corporation (Ludwigshafen, Germany). Polyethylene glycol (PEG) 400 was obtained from Kelong Chemical Corporation (Chengdu, China). Male Sprague Dawley rats (200 g ± 20 g) and nude mice (18 g ± 2 g) were supplied by the Laboratory Animal Center of Medical University (Chongqing, China).

### 2.2. Preparation and formulation of EFEN

**Figure 1.**
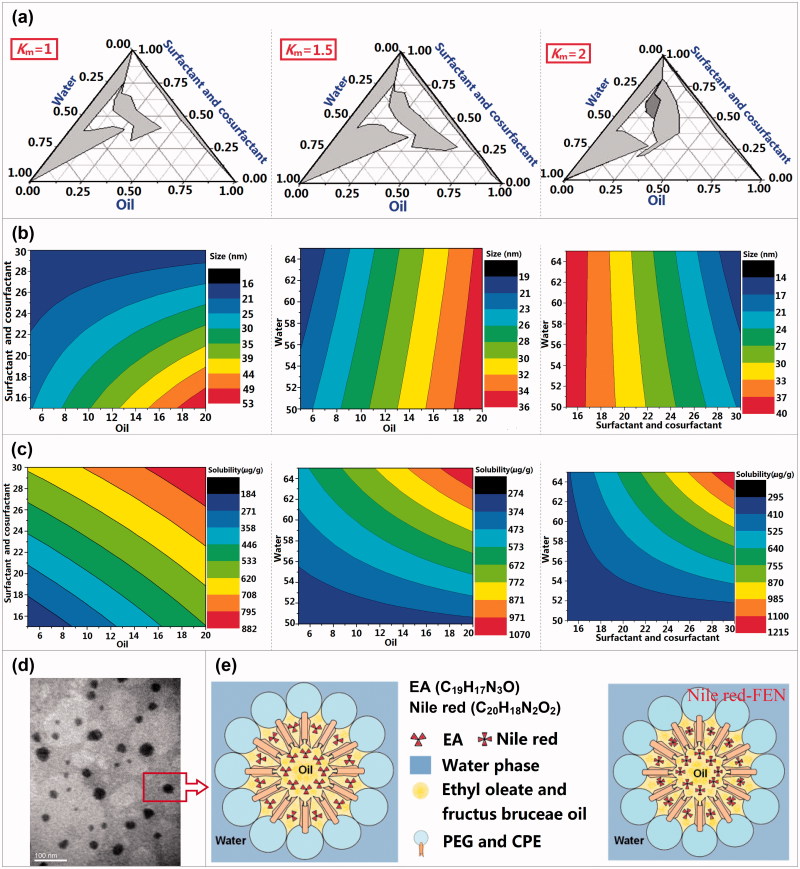
The formulation optimization, physical properties, morphologies, and structure schematic diagram of EFEN. (a) Ternary phase diagrams at the different *K*_m_ values (*K*_m_ is the mass ratio of surfactant and cosurfactant. (b) The effects of prescription components on the size and (c) solubility. (d) The transmission election photomicrograph of EFEE. (e) Schematic illustration of EFEE and Nile red-labeled FEN.

EFEN was prepared using a water titration method at ambient temperature (Yehia et al., 2017). The complex oil consisted of fructus bruceae oil and ethyl oleate (1:2, w:w). The oily phase (castor oil polyoxyethylene (35) ether (CPE) and polyethylene glycol (PEG) 400) was titrated dropwise with distilled water under gentle magnetic stirring. The compositions of three phases at every transition point between clear and cloudy interfaces were recorded to construct ternary phase diagrams (Figure 1(a)) by changing the mass ratios of CPE and PEG (1:1, 1.5:1, 2:1) and the mass ratios of the mixture of CPE and PEG to oily phase (from 9:1 to 1:9, gradually). The shaded area represents the fractional composition that is able to form emulsive nanosystems. The effects of principal components on size and solubility were investigated using the simplex lattice design method and the results were depicted using Origin Pro. 7.5 software (Origin Lab. Corp., Wellesley, MA).

### 2.3. Micromorphology, particle size, and zeta potential analysis

The micromorphology of EFEN was observed using an H-7500 transmission electron microscopy (Hitachi, Tokyo, Japan). The size and zeta-potential of EFEN were determined using a Zeta-Sizer Nano-ZS90 spectroscopy (Zeta, London, UK).

### 2.4. Endocytosis of emulsive nanosystems by tumor cells and influences of inhibitors

The uptake of emulsive nanosystems by A549 cells was investigated visually using a CLSM microscope (Leica Microsystems, Heidelberg Gmbh, Germany). The tumor cells were incubated in 24-well plates with sterile glass slides. Once the cells reached 80% confluency, medium containing different concentrations of Nile red-loaded emulsive nanosystems (NFEN) or Nile red-PBS solution (negative control) were added. At predetermined time points, the tumor cell nuclei were stained with 1.62 μM Hoechst 33342 and observed.

Quantitative cellular uptake studies were performed at 37 °C and 4 °C. A549 cells in 6-well plates were treated with 15.7 μM NFEN, incubated at 37 °C or 4 °C for 4 h, and analyzed with a FACSCaliber flow cytometer (Becton Dickinson, Franklin Lakes, NJ). Testing the effects of inhibitors on cellular uptake was performed as follows: 15.7 μM NFEN were added to A549 cells that were pretreated with 31.45 μM chlorpromazine or 200 μM genistein for 1 h. Cells were incubated for another 4 h, after which their fluorescence intensities were analyzed by flow cytometry.

### 2.5. Effect of emulsive nanosystems on cellular growth inhibition

To investigate the cytotoxicity of free EA, Blank-NE, EEN, FEN, and EFEN in A549 cells, MTT assays were performed as described previously (Fang et al., 2014). A549 cells were seeded at a density of 1 × 10^4^ cells per well in 96-well plates. After incubation overnight for adherence, the previous medium was replaced with medium containing various concentrations of free EVO, BNE, or ENE. After 12 h, 24 h, or 48 h of incubation, the medium was removed and 5 mg/mL MTT solution was added to each well. Cells were further incubated for 4 h, and the medium was replaced with 150 μL DMSO to solubilize the converted formazan. Absorbance was measured at 570 nm with a Sunrise microplate photometer (Tecan, Salzburg, Austria). The treated cells were observed using fluorescence microscopy (Olympus, Tokyo, Japan).

### 2.6. Cell-cycle arrest, apoptosis, mitochondria membrane potential, and calcium ions

Tumor cells were treated with free EA, Blank-NE, EEN, FEN, or EFEN for 24 h. For cell-cycle arrest determination, cells were then harvested using trypsin, fixed with 75% ethanol, and stained using a solution of PI, RNase A, and Triton X-100. For apoptosis determination, cells were stained with a solution of PI and Annexin V-FITC. After treatment, cells were analyzed by a FACSCaliber flow cytometer equipped with CellQuest software (Becton Dickinson, Franklin Lakes, NJ). The mitochondrial membrane potential and intracellular calcium ion levels were measured by staining with JC-1 and Fluo-3, respectively.

### 2.7. Expressed proteins related to cell cycle and apoptosis

The cyclin B, cell division cycle (CDC) 2, cyclin A, CDC 1, caspase-3, caspase-8, caspase-9, Bcl-2, and Bax protein levels were determined by Western blotting. The tumor cells were treated with free EA, Blank-NE, EEN, FEN, or EFEN for 24 h. The proteins were analyzed using primary and secondary antibodies (rabbit anti-cyclin B, anti-cyclin A, anti-CDC2, and anti-CDC1; mouse anti-CDC2 and anti-CDC1; rabbit anti-caspase-3, anti-caspase-9 and anti-Bcl-2; mouse anti-caspase-8, mouse anti-Bax, anti-rabbit HRP-IgG, and anti-mouse HRP-IgG, in 3% BSA) (Fang et al., 2014).

### 2.8. *In vivo* kinetic, bioavailability, and distribution characteristics

The male rats were orally given EFEN or EA at the same 100 mg/kg dose. Venous blood samples were collected and separated by centrifugation at 3000 rpm for 10 min and analyzed by HPLC (Tan et al., 2012). The relative bioavailability of EFEN was obtained by dividing the EFEN area under concentration-time (*AUC*) with that of EA. The tumor cells (2 × 10^7^) were injected subcutaneously into the left axilla of the nude mice. After 14 d, NFEN was injected subcutaneously into the area near the visible tumor and observed under a Maestro™ EX-RRO (CRI corp., USA).

### 2.9. Anticancer effects

The nude C57BL/6J mice bearing A549 lung cancer cells were randomly divided into four groups. Three groups were given with EFEN, blank EFEN, or EA at the same dose (8 mg/kg) via intraperitoneal route once a day for 14 d, respectively. The control group was administered with saline water. The tumor sizes and weight were measured on day 15.

### 2.10. Preliminary stimulation and hemolysis investigation

The quadriceps femorises of rabbit thighs were intramuscularly injected with EFEN to test stimulation. The quadriceps femoris was split lengthwise and observed. Hemolysis was carried out using rabbit erythrocyte suspension (5%) according to the previously described method by our team (Xiong et al., 2015). EFEN was added to the erythrocyte suspension. The mixture was placed at 37 °C for 2 h and images were acquired.

### 2.11. Statistical analysis

Results were expressed as mean ± S.D. of at least three independent experiments. Student’s *t*-test was used to compare the mean of each group with that of the control group and *p* ＜ .05 was considered significant different. The pharmacokinetic and bioequivalence analyses were conducted using Drug and Statistics software (Mathematical Pharmacology Professional Committee of China, Shanghai, China).

## 3. Results and discussion

### 3.1. Prescription, preparation, and characteristics

EFEN (containing 4.6 mg/mL EA) was prepared using a water titration method. Fructus bruceae oil and ethyl oleate served as the oil phase. CPE and PEG 400 were used as a surfactant and a cosurfactant, respectively. The formulation was optimized according to the nanoemulsion size and amount of loaded EA content in EFEN. As shown in the ternary phase diagrams (Figure 1(a)), areas capable of forming emulsive nanosystems changed along with changes in the mass ratio of surfactant and cosurfactant. The relationships of size values and preparation component ratios were calculated using the Origin Pro 7.5 software (Figure 1(b,c)). The regression equations were listed as follows:

(1) $$Y_{\text{1}}=\text{15}.\text{4897}+\text{1}.\text{1553}X_{\text{1}}+0.\text{7}0\text{75}X_{\text{2}}+0.00\text{71}X_{\text{3}}-0.0\text{296}X_{\text{1}}X_{\text{2}}+0.0\text{433}X_{\text{1}}X_{\text{3}}-0.0\text{125}X_{\text{2}}X_{\text{3}}-0.00\text{15}X_{\text{1}}X_{\text{2}}X_{\text{3}}(R^{\text{2}}=0.\text{9959},p<.000\text{1})$$

(2) $$Y_{\text{2}}=\text{1423}.\text{8}0\text{48}+\text{114}.\text{4165}X_{\text{1}}-\text{68}.\text{4178}X_{\text{2}}-\text{31}.\text{85}0\text{6}X_{\text{3}}-\text{9}.\text{4572}X_{\text{1}}X_{\text{2}}-\text{1}.\text{4778}X_{\text{1}}X_{\text{3}}+\text{1}.\text{7438}X_{\text{2}}X_{\text{3}}+0.\text{1571}X_{\text{1}}X_{\text{2}}X_{\text{3}}\left(R^{\text{2}}=0.\text{7746},p<.0\text{5}\right)$$

where the *Y*_1_ and *Y*_2_ values referred to the size and the solubility, respectively; the *X*_1_, *X*_2_, and *X*_3_ values referred to the percent contents in oil phase, sum of surfactant and cosurfactant, and water phase, respectively.

The EFEN was sphere-like in shape (Figure 1(d)). The size was uniform and determined to be 31.21 ± 0.91 nm with a polydispersity index of 0.20 ± 0.03 using a Zetasizer (Zeta, London, UK). The small droplet size and the spherical shape of nanocarriers suggested large oil/water interfaces, which have been reported to be essential for the interaction of droplets with cell membranes and likely result in efficient cellular trafficking of drugs (Sahay et al., 2010).

Most antitumor ingredients found in nature are poorly soluble. These active components include alkaloids, such as EA, capsaicin and quinine; terpenes, such as paclitaxel, tanshinone IIA and ginsenoside Rb1; acids, such as salvianolic acid B; and coumarins, such as dicoumarol (Fong et al., 2013). These poorly soluble substances are usually expected to have much better absorption and higher bioavailability. It is essential to develop delivery systems to effectively carry these poorly soluble drugs to targeted areas. In this paper, we report a novel woody oil-based emulsive nanosystem to effectively carry the alkaloid EA to tumor cells. This kind of nanosystem may also be useful to carry other poorly soluble natural drugs.

Compared with solid nanosystems, emulsive nanosystems have better fluidity, which makes it much easier to carry poorly soluble drugs to the targeted area. Compared to normal emulsive nanosystems, some specific vegetable oil-based emulsive nanosystems had an additional component: a woody oil such as fructus bruceae oil (Nie et al., 2012) or a gramineous seed oil such as coix seed oil. These specific vegetable oils can also serve as antitumor drugs and their function can be improved by advanced drug formulations. For example, fructus bruceae oil can be delivered via spongosome to treat A549 lung cancer cells (Zou et al., 2017). Furthermore, the anti-ovarian cancer activity of fructus bruceae oil was enhanced by being loaded into luteinizing hormone-releasing hormone receptor-targeted liposomes (Ye et al., 2016). In addition, coix seed oil can be carried by octanoyl galactose ester-modified intragastrical microemulsion for enhanced tumor targeting and hepatoma therapy (Qu et al., 2017). Fructus bruceae oil and coix seed oil loaded in an intravenous emulsion could be administered simultaneously for synergic antitumor application (Yu et al., 2008).

Specific vegetable oils have also been used to formulate different kinds of carriers to deliver various antitumor drugs. For example, coix seed oil was used to form microemulsions to deliver etoposide and ginsenoside Rh2 for drug-resistant breast cancer treatment via oral administration (Qu et al., 2017). In this study, fructus bruceae oil was used to form emulsive nanosystems to carry EA for more effective tumor cell uptake and enhanced bioavailability. Fructus bruceae oil played three roles: the first was as an oil phase, the second was as an antioxidant (also called a stabilizer, since this oil mainly contained polyunsaturated fatty acid), and the third was as an antitumor drug. The oil had two excipient-like properties to contribute to the formulation and one drug-like property that exerted synergistic antitumor effects.

### 3.2. Qualitative and quantitative cellular uptake studies

**Figure 2.**
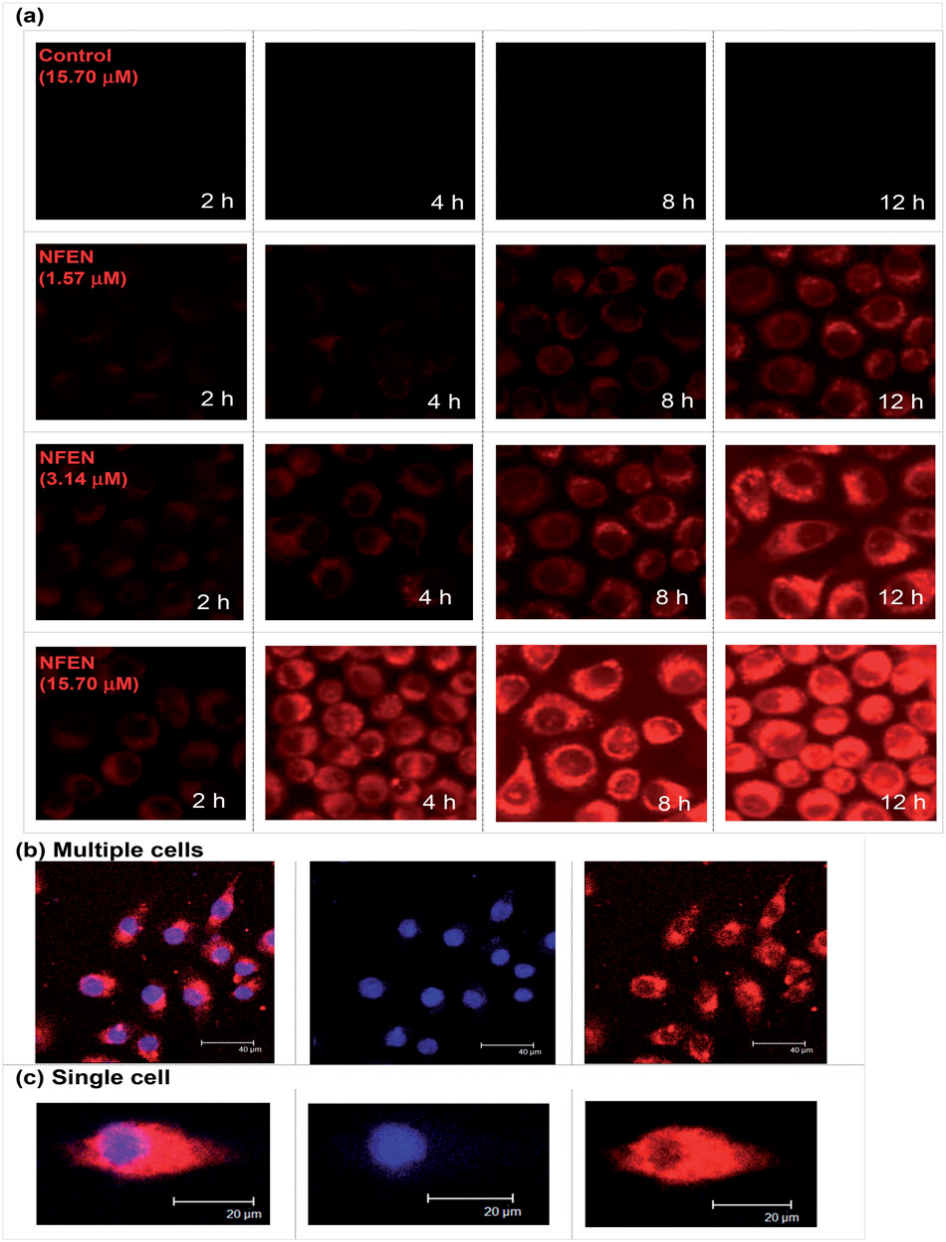
Cellular uptake of emulsive nanosystems by the A549 cells at different timepoints. (a) Confocal laser scanning photomicrographs of A549 cell uptake of emulsive nanosystems, such as (b) multiple cells and (c) single cell uptake.

**Figure 3.**
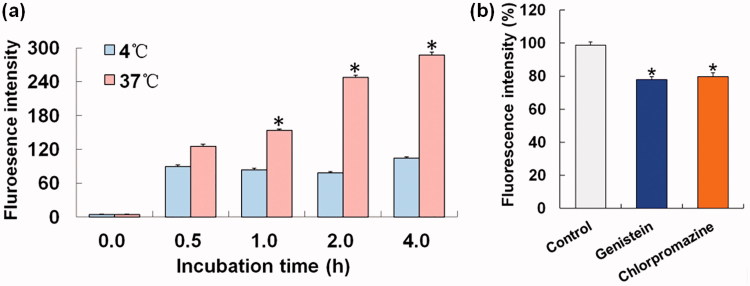
Effects of energy and inhibitors on the NFEN endocytosis by A549 cells. (a) Cellular endocytosis situations at 4 °C and 37 °C; (b) cellular endocytosis inhibited by genistein and chlorpromazine. Results were presented as the mean ± standard deviation (*n* = 3). *p ＜ .05 for the test sample compared with the other group (a) or the control (b).

As shown in Figure 2, Nile red-loaded emulsive nanosystems (NFEN) entered tumor cells in a time- and concentration-dependent manner within 12 h. Red fluorescence in the cytoplasm intensified with increase in incubation duration or in concentration. NFEN moved swiftly and randomly in the cytoplasm and tended to accumulate near the cell nuclei. The cellular uptake of Nile red-PBS solution (negative control) by tumor cells was too low to be visualized by fluorescence microscopy. As shown in Figure 3(a), the fluorescence intensity of NFEN at 37 °C significantly intensified over 4 h and was much higher than the intensity at 4 °C at the corresponding time point. Compared with the control, the fluorescence intensities of cells treated with genistein and chlorpromazine decreased to similar extents (22% versus 20%) (Figure 3(b)).

Cellular trafficking (or endocytosis) of nanomedicines was closely related not only to its components, shape, structure and physico-chemical characteristics but also to the cell types (Sahay et al., 2010). In this paper, A549 cells were selected as a model cell line to study the endocytosis of EFEN for multiple reasons. First, EA had no biological activity in A549 cells in our preliminary experiments (data not show), and application of this effective, systemic and specific (seemingly only cytotoxic to cancer cells) anti-tumor agent on A549 cells was an attempt to finally offer evidence for its clinical utilization in the treatment of NSCLC, a leading devastating life-killer (Liu et al., 2017). Second, A549 cells, a human alveolar epithelial cell line, can be used to mimic the adsorption of lung cancer drug delivery nanosystems *in vitro*, which is an appealing area of study for nanomedicine (Ikari et al., 2015). Finally, understanding endocytosis in nanomedicine delivery is essential for the development of lung cancer drug delivery nanosystems that target transport proteins.

Both qualitative and quantitative cellular uptake studies proved that incorporating EA into emulsive nanosystems improved its cellular uptake. The analyses showed that EFEN (∼337 μg/mL) had a higher solubility than EA (∼3 μg/mL), which made it more likely to enter the cells. EFEN also had a small diameter (∼30 nm), which made it more likely to attach to and enter the cells (Edelman et al., 2017). In addition, EFEN had a lower negative zeta potential (∼−1 mV), which caused it to experience less repulsion force from the negatively charged cell membrane. It has been reported that nanosystems are sometimes designed as cationic formulations in order to decrease repulsion (Liu et al., 2016). EFEN enhanced the mobility through cellular membranes by using liquid emulsion nanosystems that contained liquid components such as oil and surfactants (Ganta et al., 2014).

The cellular uptake of EFEN by tumor cells was a dose-dependent and time-dependent process over 12 h. Furthermore, it was an energy-dependent process since much more EFEN was taken into cells at body temperature than at lower temperature. Pinocytosis refers to a process in which liquid droplets are ingested by living cells. Since it is in the fluid state, EFEN might be taken into tumor cells via pinocytosis. Chlorpromazine is a clathrin-mediated pathway inhibitor, while genistein is both caveolae-dependent and clathrin-independent pathway inhibitor. Chlorpromazine and genistein were separately applied to investigate the transport proteins involved in the endocytosis procedure. The decreased uptaken amount of tumor cells after adding two inhibitors suggested that NFEN was trafficked through three pinocytosis pathways: clathrin-mediated, caveolae-dependent, and clathrin-independent pathways. This is consistent with previous reports that microbubbles induced clathrin-mediated endocytosis and fluid-phase uptake through distinct mechanisms (Fekri et al., 2016) and that doxorubicin-conjugated poly(methacrylic acid-*co*-cholesteryl methacrylate) copolymers were internalized through both clathrin-dependent and clathrin-independent endocytosis mechanisms (Sevimli et al., 2015). However, octanoyl galactose ester-modified microemulsion systems containing coix seed components were reported to improve accumulation in tumors through asialoglycoprotein receptor-mediated endocytosis (Qu et al., 2017).

### 3.3. Inhibitory effects of EFEN on lung tumor cell growth

**Figure 4.**
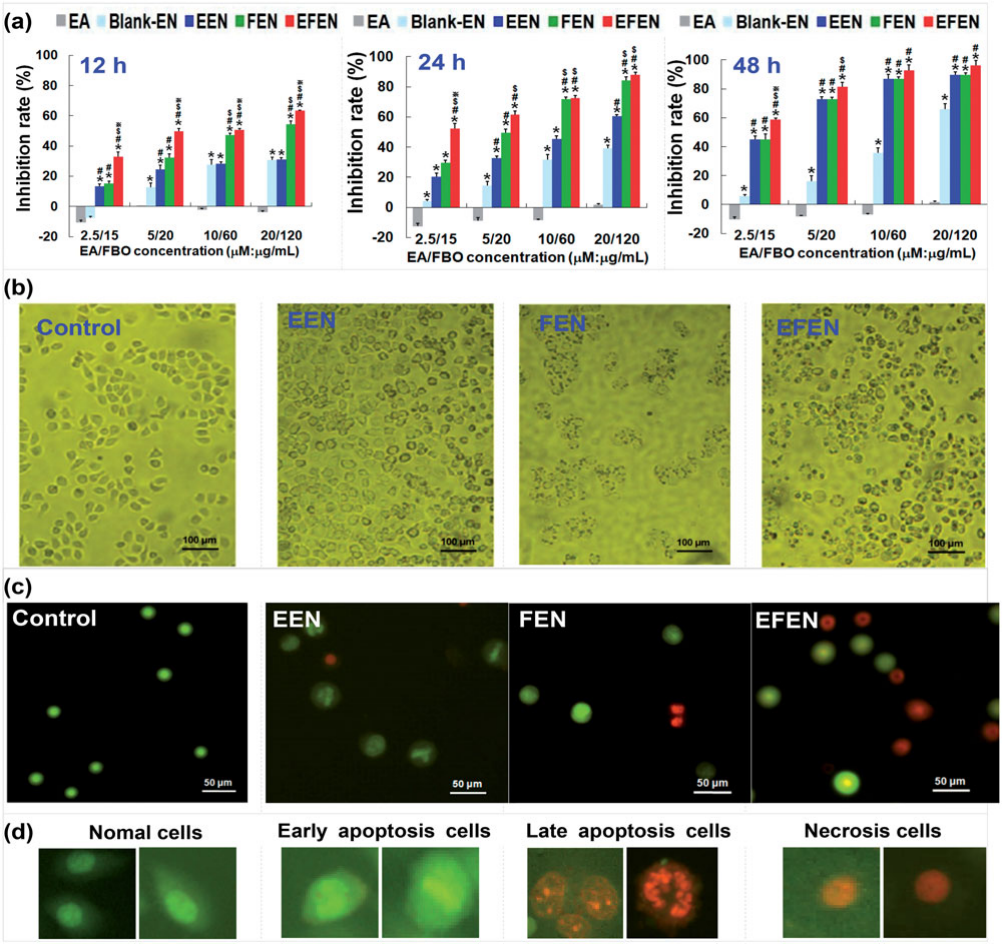
Effects of EA and EFEN on the cell growth, cell phase and apoptosis of lung cancerous cells. (a) Inhibition rates of the A549 cells treated with free EA and EFEN for 12 h, 24 h, and 48 h, respectively. Results were presented as the mean ± standard deviation (*n* = 3), *p ＜ .05 for the test sample compared with EA, #*p* ＜ .05 for the test sample compared with blank EN, $*p* ＜ .05 for the test sample compared with EEN, ※*p* ＜ .05 for the test sample compared with FEN. Morphologies of (b) A549 cells and (c) AO/EB double staining cells without treatment (i.e. normal A549 cells) or treated with EEN, FEN and EFEN. (d) Typical morphologies of AO/EB double staining cells in different apoptosis stages.

As shown in Figure 4(a,b), EFEN exhibited inhibitory effects in a time-dependent manner. The half-maximal inhibition concentrations (IC_50_) of EFEN were 6.79 μM, 1.95 μM, and 1.55 μM for 12 h, 24 h, and 48 h, respectively. Moreover, EFEN also exhibited inhibitory effects in a dose-dependent manner. The rates of suppression of tumor cells treated with EFEN for 24 h increased from 33% to 63% when EA concentrations increased from 2.5 μM to 20 μM.

The molecular basis for anti-tumor activities exerted by EA in A549 cells was explored. A G2/M phase blockage and apoptosis induction occurred in tumor cells treated with EFEN (Figure 5(a,b)). After 24 h incubation with 2 μM EFEN, the proportion of cells arrested in G2/M was 30.87 ± 1.96% compared with 10.23 ± 2.84% after treatment with free EA. The apoptosis rate was 17.97 ± 2.35% compared with 8.81 ± 1.16% by free EA. Compared with free EA treatment, EFEN resulted in lower mitochondrial membrane potential and higher intracellular calcium ion concentration. The morphologies of tumor cells treated with 2 μM EFEN were observed by AO/EB double staining (Figure 4(c,d)) (Liu et al., 2017). After treatment with EFEN, tumor cells in early apoptosis showed concentrated green, which indicated chromatin condensation; cells in late apoptosis showed discrete orange, which indicated the presence of apoptosis bodies; and cells undergoing necrosis showed concentrated orange dye. After treatment with EFEN, the intracellular calcium ion concentration increased, and this was followed by a decline in the mitochondrial membrane potential of tumor cells (Figure 5(c,d)) (Tan et al., 2013).

Figure 5.Effects of EA and EFEN on (a) cell phase, (b) apoptosis, (c) mitochondrial membrane potential, (d) intracellular calcium ion, and (e) protein levels of cyclin B, CDC 2, cyclin A, and CDC 2. (f) Protein levels of caspases-3, -8, -9, Bcl-2, and Bax of lung cancerous cells. Results were presented as the mean ± standard deviation (*n* = 3). Results were presented as the mean ± standard deviation (*n* = 3), *p ＜ .05 for the test sample compared with EA, #*p* ＜ .05 for the test sample compared with Blank EN, $*p* ＜ .05 for the test sample compared with EEN, ※*p* ＜ .05 for the test sample compared with FEN.

**Figure:**
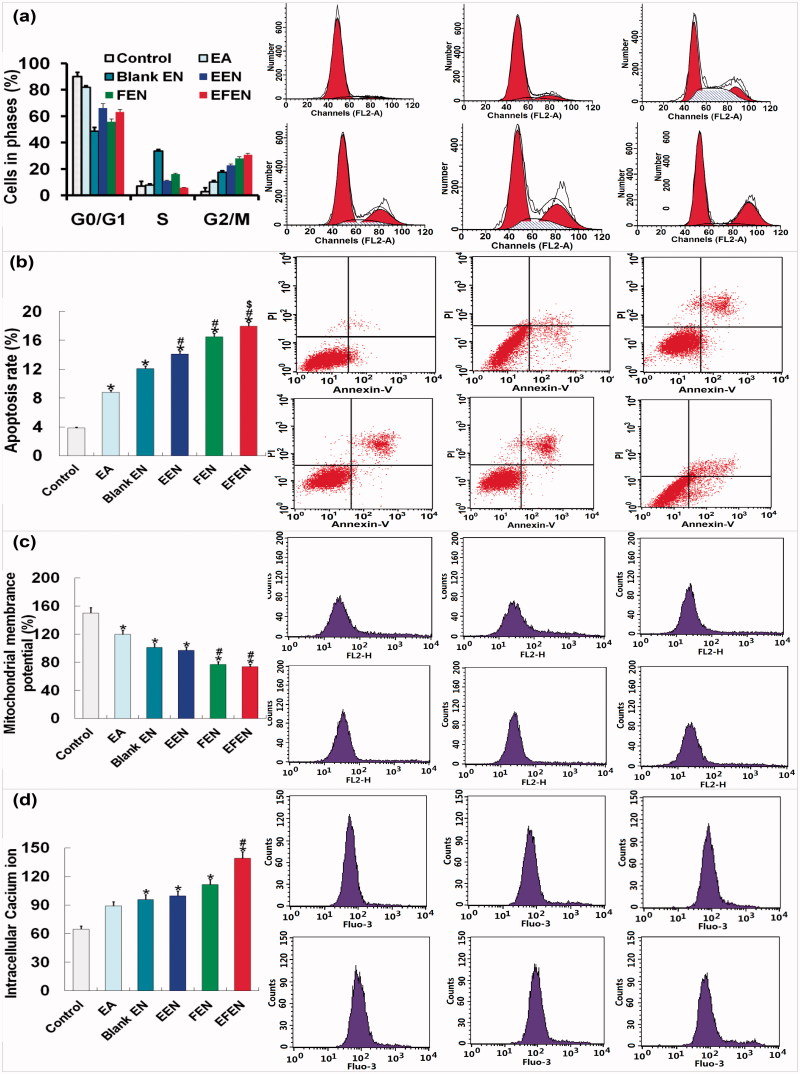

**Figure:**
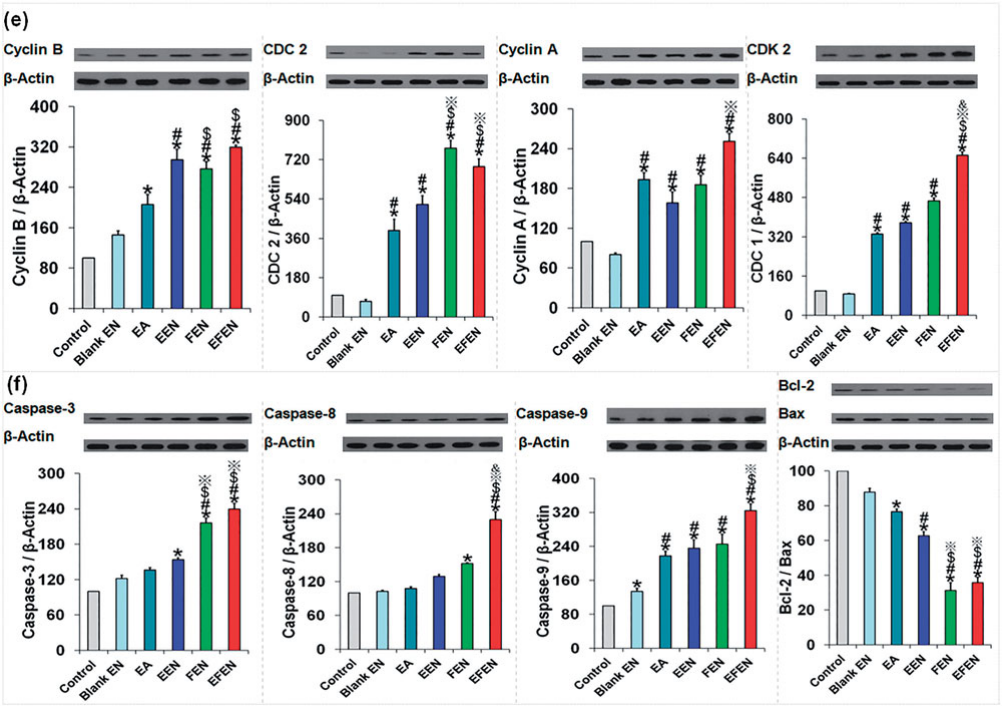

Compared with free EA treatment, EFEN-treated cells had higher protein expression of cyclin B and cell division cycle-regulated protein 2 (Figure 5(e)). EFEN might cause mitosis or division lag via activation of cyclin B/CDC 2. Compared with free EA, EFEN-treatment resulted in higher protein expression of caspase-3, -8, and -9, and lower protein levels of Bcl-2/Bax (Figure 5(f)).

The anti-tumor activity of EFEN was mediated by the inhibition of cell viability, the induction of apoptosis and cell cycle arrest at the protein level. EFEN might induce apoptosis through intrinsic and extrinsic caspase-dependent pathways. Our findings suggested that EFEN treatment up-regulated CDC2/cyclin B levels and further induced G2/M arrest and that EFEN induced apoptosis by up-regulating Bcl-2/Bax ration and activating caspase-3, -8 and -9. Thus, EFEN induced apoptosis through diverse caspase-dependent pathways (Park et al., 2017). More work should be done to classify in more detail the apoptotic pathways involved. For example, pan-caspase inhibitors can be employed to block the caspase-dependent pathway, or translocation of apoptosis-inducing factor into nucleus can be studied for a direct investigation of caspase-independent pathways.

### 3.3. *In vivo* kinetic, bioavailability, and distribution characteristics

**Figure 6.**
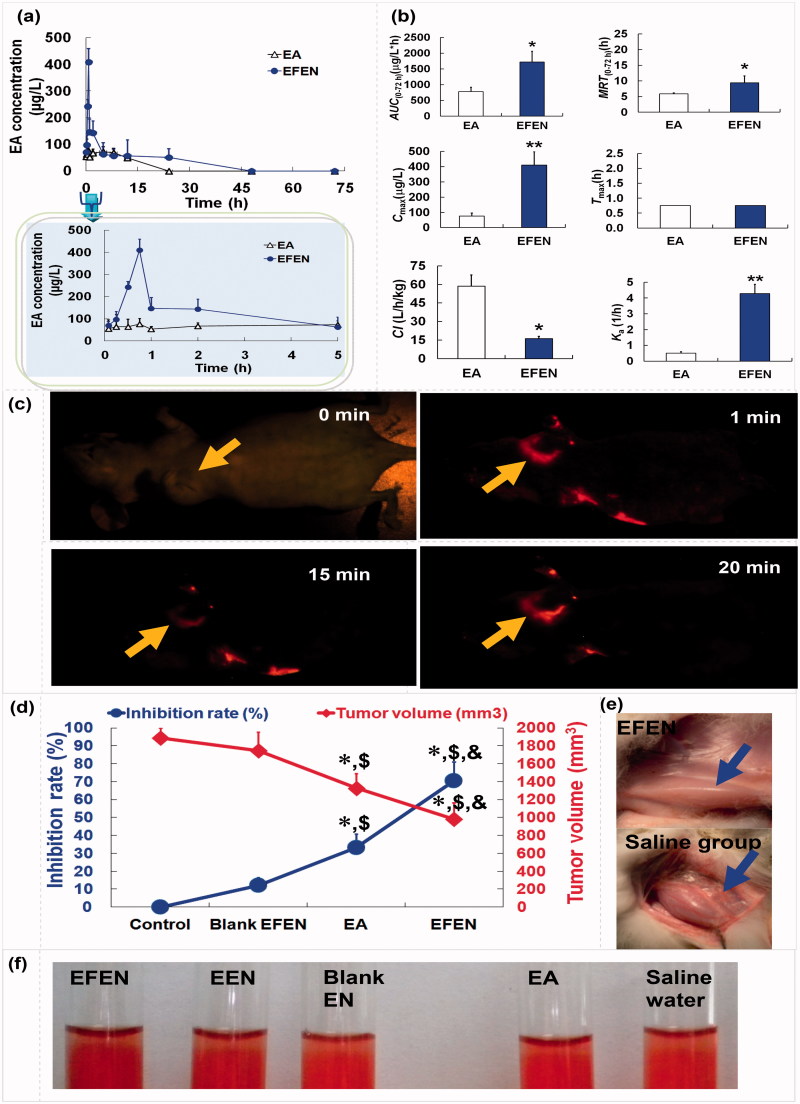
The *in vivo* kinetic, distribution characteristics, anticancer effects, and safety of EA and EFEN. (a) Plasma EA concentration versus time profiles; (b) pharmacokinetic parameters of EA and EFEN. The data were shown as mean ± SD. *n* = 6 rats per group. *p ＜ .05 indicated significant differences between EA and EFEN; (c) accumulation of EFEN at the tumor site after administration; (d) effects of EFEN on cancer sizes and weight, *p ＜ .05 indicated significant differences between the sample group and the control group, $P < .05 indicated significant differences between the sample group and Blank EFEN group, & P ＜.05 indicated significant differences between the sample group and EA; (e) stimulation; and (f) hemolytic evaluations of EFEN. Normal saline solution was used as the negative control in stimulation and hemolytic tests.

EFEN markedly improved the absorption and availability of EA, resulting in a higher absorptive constant (8.38 times) and higher bioavailability (362.21% increase) (Figure 6(a,b)). NFEN was retained in the tumor area when injected subcutaneously into the tissue near the tumor (Figure 6(c)).

EFEN had better pharmacokinetic behavior than EA alone. The higher bioavailability was related to higher absorption, higher concentration over time, and lower clearance. The superior pharmacokinetic properties of EFEN definitely favored the production of therapeutic effects (Zhou et al., 2016). EFEN could be maintained in the tumor area via injection.

### 3.4. Preliminary evaluation of the anticancer effects and safety

Compared with the negative control, both EFEN and EA had obvious antitumor effects (Figure 6(d)). In addition, compared with free EA, the EFEN group had slower tumor growth evidenced by smaller tumor size and lower tumor weight. There is significant difference between the EFEN group and the control group, the EA group and the control group, the EFEN group and the EA group. Above results suggested superior antitumor effects of EFEN.

Rabbits administered EFEN had a zero-order stimulative reaction, meaning no changes were observed (Figure 6(e)). EFEN also produced no hemolysis (Figure 6(f)). In addition, it was safe to inject tissues with EFEN. Preliminary stimulation and hemolytic evaluations suggested its safety (Zhang et al., 2005).

## 4. Conclusions

Most bioactive ingredients from nature have low-solubility. To achieve better absorption and higher bioavailability, we first formulated the woody oil-based emulsive nanosystem using fructus bruceae oil to deliver the antitumor agent evodiamine (EFEN). In addition to the role of synergistic antitumor drug, fructus bruceae oil played two additional roles as the oily phase and emulsion stabilizer in EFEN. EFEN exhibited superior anti-tumor activities in NSCLC A549 cells when compared with free EA, a blank nanoemulsion, an EA-loaded emulsive nanosystem, and a fructus bruceae oil-loaded emulsive nanosystem. These effects were mediated by the inhibition of cell viability, the induction of apoptosis and cell cycle arrest at the protein level. The increased sensitivity of EFEN may be related to the enhanced cellular uptake of EFEN via multiple pathways, as well as the favorable increases in bioavailability and drug persistence in the tumor area. For the first time, increased sensitivity of lung cancer cells to poorly soluble natural alkaloids was able to be achieved by delivering drugs with woody oil-based emulsive nanosystems. EFEN has potential as an effective anti-tumor treatment. Woody oil-based emulsive nanosystems may deliver poorly soluble natural alkaloids efficiently.
